# Neuropathologic Features of Antemortem Atrophy-Based Subtypes of Alzheimer Disease

**DOI:** 10.1212/WNL.0000000000200573

**Published:** 2022-07-26

**Authors:** Rosaleena Mohanty, Daniel Ferreira, Simon Frerich, J-Sebastian Muehlboeck, Michel J. Grothe, Eric Westman, on behalf of the Alzheimer's Disease Neuroimaging Initiative

**Affiliations:** From the Division of Clinical Geriatrics (R.M., D.F., S.F., J.S.M., E.W.), Department of Neurobiology, Care Sciences and Society, Karolinska Institutet, Stockholm, Sweden; Department of Radiology (D.F.), Mayo Clinic, Rochester, MN; Institute for Stroke and Dementia Research (E.W.), University Hospital, Ludwig-Maximilian-University (LMU) Munich, Germany; Unidad de Trastornos del Movimiento (M.J.G.), Servicio de Neurología y Neurofisiología Clínica, Instituto de Biomedicina de Sevilla, Hospital Universitario Virgen del Rocío/CSIC/Universidad de Sevilla, Spain; Clinical Dementia Research Section (M.J.G.), German Center for Neurodegenerative Diseases (DZNE), Rostock, Germany; and Department of Neuroimaging (E.W.), Centre for Neuroimaging Sciences, Institute of Psychiatry, Psychology and Neuroscience, King's College London, UK.

## Abstract

**Background and Objectives:**

To investigate whether antemortem MRI-based atrophy subtypes of Alzheimer disease (AD) differ in neuropathologic features and comorbid non-AD pathologies at postmortem.

**Methods:**

From the Alzheimer's Disease Neuroimaging Initiative cohort, we included individuals with antemortem MRI evaluating brain atrophy within 2 years before death, antemortem diagnosis of AD dementia/mild cognitive impairment, and postmortem-confirmed AD neuropathologic change. Antemortem atrophy subtypes were modeled as continuous phenomena based on a recent conceptual framework: typicality (spanning limbic-predominant AD to hippocampal-sparing AD) and severity (spanning typical AD to minimal atrophy AD). Postmortem neuropathologic evaluation included AD hallmarks, β-amyloid, and tau as well as non-AD pathologies, alpha-synuclein and TAR DNA-binding protein 43 (TDP-43). We also investigated the overall concomitance across these pathologies. Partial correlations assessed the associations between antemortem atrophy subtypes and postmortem neuropathologic outcomes.

**Results:**

In 31 individuals (26 AD dementia/5 mild cognitive impairment, mean age = 80 years, 26% females), antemortem typicality was significantly negatively associated with neuropathologic features, including β-amyloid (rho = −0.39 overall), tau (rho = −0.38 regionally), alpha-synuclein (rho = −0.39 regionally), TDP-43 (rho = −0.49 overall), and concomitance of pathologies (rho = −0.59 regionally). Limbic-predominant AD was associated with higher Thal phase, neuritic plaque density, and presence of TDP-43 compared with hippocampal-sparing AD. Regionally, limbic-predominant AD showed a higher presence of tau and alpha-synuclein pathologies in medial temporal structures, a higher presence of TDP-43, and concomitance of pathologies subcortically/cortically compared with hippocampal-sparing AD. Antemortem severity was significantly negatively associated with concomitance of pathologies (rho = −0.43 regionally), such that typical AD showed higher concomitance of pathologies than minimal atrophy AD.

**Discussion:**

We provide a direct antemortem-to-postmortem validation, highlighting the importance of understanding atrophy-based heterogeneity in AD relative to AD and non-AD pathologies. We suggest that (1) typicality and severity in atrophy reflect differential aspects of susceptibility of the brain to AD and non-AD pathologies; and (2) limbic-predominant AD and typical AD subtypes share similar biological pathways, making them more vulnerable to AD and non-AD pathologies compared with hippocampal-sparing AD, which may follow a different biological pathway. Our findings provide a deeper understanding of associations of atrophy subtypes in AD with different pathologies, enhancing the prevailing knowledge of biological heterogeneity in AD and could contribute toward tracking disease progression and designing clinical trials in the future.

Alzheimer disease (AD) is pathologically defined by the hallmarks of β-amyloid (Aβ) plaques and tau neurofibrillary tangles (NFTs). However, pure AD is increasingly recognized as not being the most prevalent form of the disease.^1-3^ Concomitant forms of pathologic proteins such as α-synuclein (α-syn) and TAR DNA-binding protein 43 (TDP-43) have been reported in over 40%^4^ and 50%^5^ of the AD cases, respectively.

Does this multimorbid view of the brain in AD suggest that atrophy may be downstream to not only the AD hallmark pathologies but also the interactions with 1 or more concomitant pathologies? Examination of medial temporal atrophy measured on antemortem MRI in relation to postmortem neuropathology has shown that tau pathology was associated with posterior hippocampal atrophy, whereas TDP-43 pathology was associated with anterior medial temporal atrophy.^6^ Medial temporal atrophy, although a common characteristic, is not always observed in AD. Converging evidence suggests that biological heterogeneity in AD may manifest as distinct atrophy subtypes: typical AD, limbic-predominant AD, hippocampal-sparing AD, and minimal atrophy AD,^7^ with the last 2 showing relatively preserved medial temporal gray matter structure. Thus, revising the initial question, we ask: does this multimorbid view of the brain in AD suggest that atrophy subtypes may be downstream to not only the AD hallmark pathologies but also the interactions with 1 or more concomitant pathologies? To our knowledge, the answer to this question is yet to be explored.

We currently lack in vivo biomarkers to assess pathologies such as α-syn and TDP-43. Therefore, we investigated the relationship between antemortem MRI-based atrophy subtypes and postmortem neuropathologic profiles in AD. Our key research questions are (1) whether antemortem atrophy subtypes of AD are related to individual and/or concomitance of AD and non-AD pathologies at postmortem, and (2) whether this subtype-to-pathology relationship varies by brain region. Corresponding to these research questions, we hypothesized that antemortem atrophy subtypes of AD may be differentially associated with different AD and non-AD pathologies assessed postmortem, which may vary by brain region.

## Methods

### Participants

Participants were selected from the Alzheimer's Disease Neuroimaging Initiative (ADNI) database (PI: M. Weiner, launched 2003; adni.loni.usc.edu/). The goal of the ADNI is to test and use biomarkers, clinical, and neuropsychological assessments to track disease progression in AD. We included data from participants who had antemortem MRI and postmortem neuropathologic assessments (Version 11, April 12, 2018). eFigure 1, links.lww.com/WNL/C4, shows the selection criteria for this study. Our final cohort comprised 31 participants with intermediate or high AD neuropathologic change (ADNC) at postmortem examination (i.e., pathology-confirmed AD dementia; low ADNC is not an adequate explanation for cognitive impairment or dementia)^8^ and availability of an antemortem MRI scan within 2 years before death (for a more accurate antemortem approximation of the postmortem/final subtype of an individual and to avoid long antemortem-to-postmortem interval being a potential confound).

### Standard Protocol Approvals, Registrations, and Patient Consents

All the ADNI protocols were approved by the institutional review boards of each participating institution. All participants provided written informed consent in accordance with the Declaration of Helsinki.

### Antemortem Neuroimaging and Cognition

MRI scans were acquired on 1.5T or 3T scanners with T1-weighted sagittal 3D magnetization-prepared rapid gradient-echo sequences (detailed ADNI imaging protocols: adni.loni.usc.edu/methods/). MRI scans were processed cross-sectionally using FreeSurfer 6.0.0 (freesurfer.net/), automated through theHiveDB system.^9^ Resulting segmentations were visually screened for quality control. Screened scans were included for subsequent analyses. Automatic region of interest parcellation yielded volumes of 41 cortical and subcortical areas^10,11^ per hemisphere, serving as a measure of brain atrophy. We used Mini-Mental State Examination (MMSE),^12^ Clinical Dementia Rating (CDR), and composite scores for memory (ADNI-MEM)^13^ and executive function (ADNI-EF)^14^ corresponding to the MRI visit as the main outcomes to evaluate the level of cognitive impairment.

### Antemortem Atrophy Subtypes

Following the recently proposed conceptual framework for AD subtypes,^7^ we quantified MRI-based atrophy subtypes in terms of 2 principal dimensions: typicality and severity. Given the limited sample size, we modeled atrophy subtypes on a continuous scale for greater sensitivity^15^ rather than categorizing individuals into subgroups or categorical subtypes. Typicality was proxied by the ratio of hippocampal volume to whole cortical volume (ratio henceforth referred to as H:C), similar to the index adopted by the original neuropathologic subtyping study.^16^ Severity was proxied by the Global Brain Atrophy Index, measured by the ratio of whole brain volume to volume of CSF^17^ (ratio henceforth referred to as BV:CSF), such that lower values of the index correspond to more atrophy (i.e., higher severity).

### Postmortem Neuropathologic Assessment

Neuropathologic assessments were conducted as part of the ADNI neuropathology core (neuropathologist: Dr. Nigel Cairns, the Knight Alzheimer's Disease Research Center, Washington University School of Medicine, St. Louis, adni.loni.usc.edu/about/#core-container).^18^ Assessments followed the NIA-AA guidelines for the neuropathologic assessment of AD^8^ (alz.washington.edu/NONMEMBER/NP/npguide10.pdf).

### Antemortem-to-Postmortem Validation Approach

We modeled MRI-based antemortem atrophy subtypes in AD as continuous phenomena^15^ of 2 orthogonal typicality and severity dimensions, following the recent conceptual framework for AD subtypes.^7^ We then examined the relationship of these dimensions to postmortem neuropathologic features, including AD (Aβ, tau) and non-AD (α-syn, TDP-43) pathologies and concomitance across them.

To investigate our first research question of whether antemortem atrophy subtypes of AD may be related to neuropathologic differences, we examined (1) established semiquantitative rating scales for AD-specific neuropathologic measures, including the Thal phase of regional distribution of Aβ (diffuse and cored) plaques (A0–A3), the Braak stage of NFT distribution (B0–B3), and the Consortium to Establish a Registry for AD scores for the density of neuritic plaques (C0–C3)^8^; and (2) the presence/absence of comorbid non-AD pathologies, including overall α-syn (Lewy body [LB]) pathology, assessed across the brainstem, limbic region, neocortex, amygdala, and olfactory bulb as per the modified McKeith criteria,^8,19^ and overall TDP-43 pathology assessed as immunoreactive inclusions (comprising any of neuronal cytoplasmic inclusion [NCI], neuronal intraneuronal inclusion, dystrophic neurite, or glial cytoplasmic inclusion) across the amygdala, hippocampus, entorhinal cortex/inferior temporal gyrus, and frontal neocortex.^20^

To investigate our second research question of whether antemortem atrophy subtypes of AD may be related to postmortem pathologies varying by brain regions, we examined regional pathologic outcomes: we analyzed regions most relevant to atrophy subtypes in AD,^7^ that is, structures of the medial temporal lobe, including the hippocampus at the level of the lateral geniculate nucleus (cornu Ammonis 1 or CA1, dentate gyrus, and parahippocampal gyrus), amygdala, and entorhinal cortex, and structures of the association cortex, including the middle frontal gyrus, superior and middle temporal gyri, and inferior parietal lobe (angular gyrus). We focused on specific forms of pathologies binarized for presence/absence: (1) AD-specific neuropathologic measures of Aβ (positive for both diffuse and cored plaques) and tau (NFT) and (2) non–AD-specific neuropathologic measures of α-syn (LB) and TDP-43 (NCI).

To investigate whether antemortem atrophy subtypes of AD may be related to concomitance of pathologies that may also vary regionally, we evaluated the total number of pathologies present per region as an outcome: each pathology was binarized for presence/absence and summed, considering both AD-specific and non–AD-specific pathologies (concomitance ranging from 0 through 4).

### Statistical Analysis

We analyzed the association between antemortem atrophy subtypes (typicality and severity as continuous independent variables in separate models) and cognition as well as neuropathologic outcomes as dependent variables using linear partial correlations, controlled for age at MRI scan and MRI scanner field strength. Furthermore, each model with typicality as an independent variable was controlled for severity and vice versa to examine whether the correlation may be solely explainable by the dimension treated as independent variable. Because of the limited sample size in this rare antemortem-postmortem dataset, we report significant results at an uncorrected *p* value of <0.05, akin to previous radiologic-pathologic association studies.^21,22^ In addition, we assessed the role of sex (binarized as female or male) and *APOE* status (categorized by all combinations of pairs of the alleles, i.e., 2-4, 3-3, 3-4, and 4-4) through mediation analyses.^23^

All statistical analyses and visualizations were conducted using MATLAB R2020b (The MathWorks, Inc., Natick, MA).

### Data Availability

Data used in this study have been made publicly available by the ADNI in the Laboratory of Neuro Imaging database.

## Results

### Participants

Table 1 shows the demographic and antemortem/postmortem characteristics of the cohort. The age at antemortem MRI was 80.0 ± 6.7 years, whereas the age at death was 81.2 ± 6.78 years. The level of cognitive impairment was higher in individuals with AD dementia than those with amnestic mild cognitive impairment (aMCI) in the cohort based on the MMSE, CDR, ADNI-MEM, and ADNI-EF. All individuals had markers of cerebrovascular disease postmortem (1 or more types of the following: macroscopic vascular brain injury, microinfarcts, microbleeds, microhemorrhages, arteriolosclerosis, white matter rarefaction, or other vascular changes).

**Table 1:**
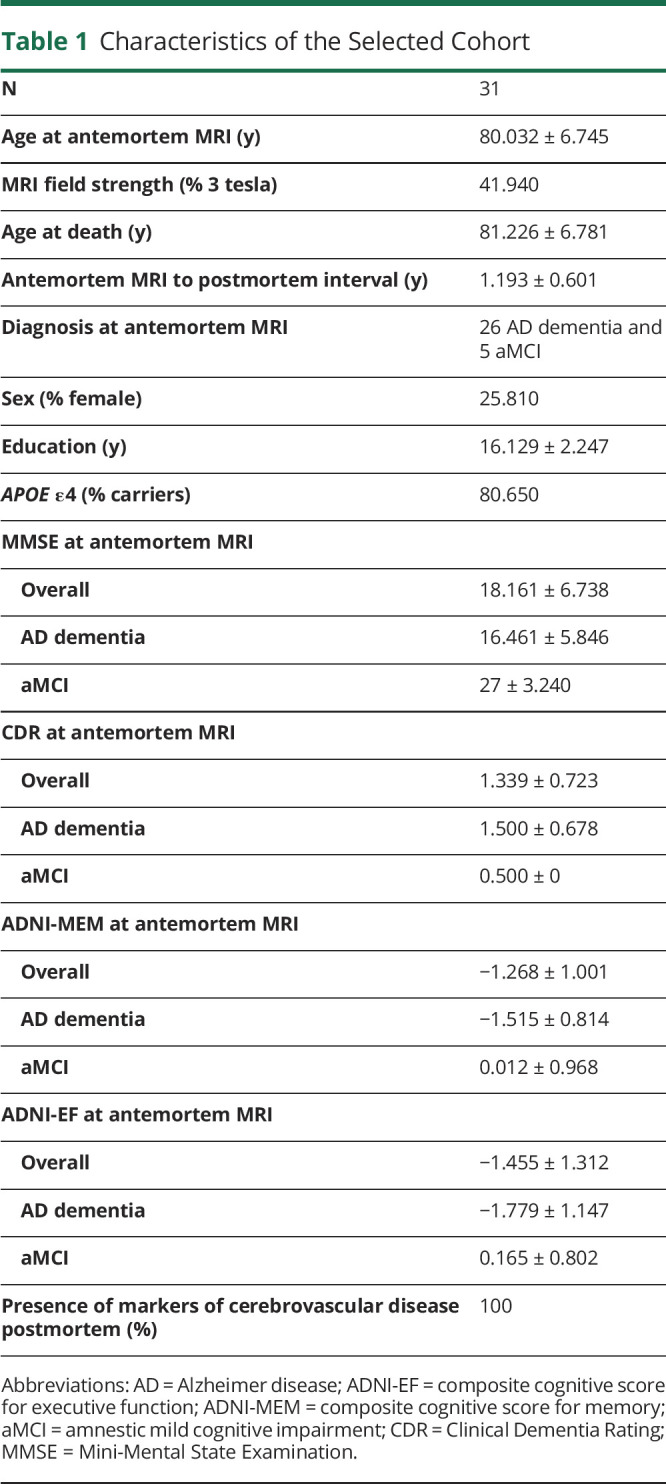
Characteristics of the Selected Cohort

### Antemortem Atrophy Subtypes

**Figure 1:**
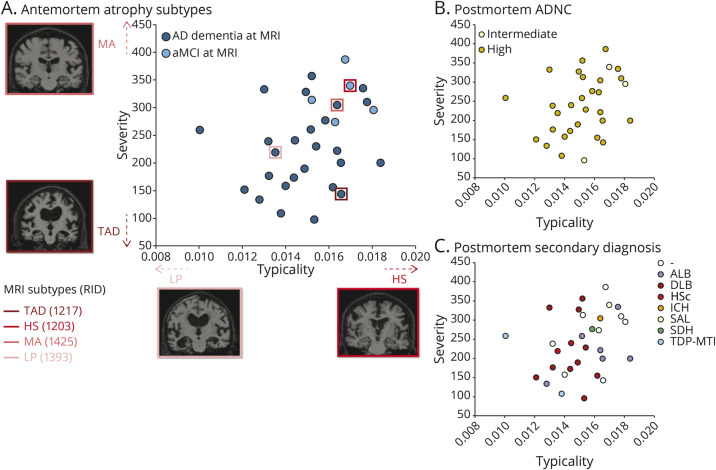
Distribution of (A) Antemortem MRI-Based Heterogeneity and (B-C) Postmortem Neuropathology Superposed on MRI-Based Heterogeneity (A) Antemortem atrophy subtypes modeled as continuous phenomena by the dimensions of typicality and severity. Four individual cases are highlighted, showing the extremes on each dimension; (B) postmortem AD neuropathologic change; and (C) postmortem secondary diagnosis assigned per individual. All plots show antemortem MRI-based typicality on the horizontal scale, proxied by the index = 
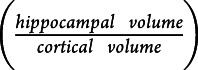
; all plots show antemortem MRI-based severity on the vertical scale, proxied by the Global Brain Atrophy Index = 
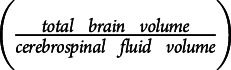
, whereby higher values correspond to lower severity. AD = Alzheimer disease; ADNC = AD neuropathologic change; ALB = amygdala Lewy bodies; aMCI = amnestic mild cognitive impairment; DLB = dementia with Lewy bodies; HS = hippocampal-sparing AD; HSc = hippocampal sclerosis; ICH = intracerebral hemorrhage; LP = limbic-predominant AD; MA = minimal atrophy AD; RID = assigned individual ID in the AD Neuroimaging Initiative dataset; SAL = subcortical arteriosclerotic leukoencephalopathy; SDH = subdural hemorrhage; TAD = typical AD; TDP-MTL = TAR DNA-binding protein in the medial temporal lobe.

Figure 1A shows the atrophy subtypes in antemortem MRI, characterized by the continuous scale measures of typicality (H:C) and severity (BV:CSF). We show 4 examples to illustrate the extremes on each dimension. On the typicality dimension, case RID 1203 represents hippocampal-sparing AD toward the higher extreme, whereas case RID 1393 represents limbic-predominant AD toward the lower extreme. Similarly, on the severity scale, case RID 1271 represents typical AD toward the lower extreme (higher severity), whereas case RID 1425 represents minimal atrophy AD toward the higher extreme (lower severity). The association between typicality and severity was not statistically significant (r = 0.3, *p* = 0.09). Antemortem severity (r = 0.5, *p* = 0.01; controlled for typicality) but not typicality (r = −0.1, *p* = 0.6; controlled for severity) was significantly associated with the MMSE.

### Association Between Antemortem Typicality and Neuropathologic Outcomes

**Figure 2:**
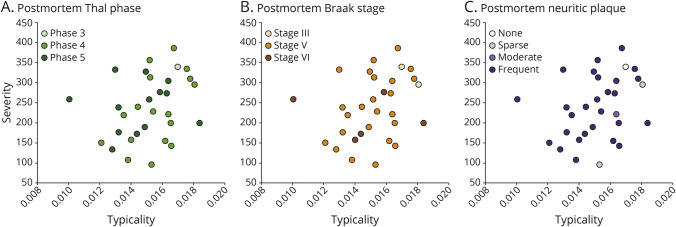
Distribution of Postmortem AD Neuropathologies Superposed on MRI-Based Heterogeneity Postmortem AD pathologies used to assess ADNC, encompassing the “ABC” scores of (A) Thal phase for Aβ, (B) Braak stage for tau, and (C) Consortium to Establish a Registry for AD neuritic plaques. All plots show antemortem MRI-based typicality on the horizontal scale, proxied by the index = 
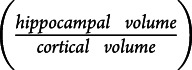
; all plots show antemortem MRI-based severity on the vertical scale, proxied by the Global Brain Atrophy Index = 
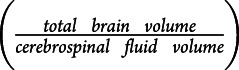
, whereby higher values correspond to lower severity. AD = Alzheimer disease; ADNC = AD neuropathologic change.

**Figure 3:**
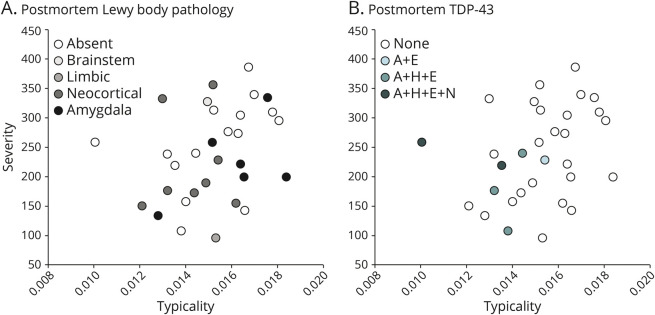
Distribution of Postmortem Non-AD Neuropathologies Superposed on MRI-Based Heterogeneity Postmortem non-AD pathologies including (A) α-synuclein Lewy bodies and (B) TDP-43. All plots show antemortem MRI-based typicality on the horizontal scale, proxied by the index = 
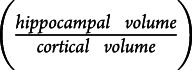
; all plots show antemortem MRI-based severity on the vertical scale, proxied by the Global Brain Atrophy Index = 
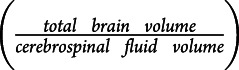
, whereby higher values correspond to lower severity. A + E = TDP-43 immunoreactive inclusions are present in the amygdala and entorhinal/inferior temporal cortex; A + H + E + N = TDP-43 immunoreactive inclusions are present in the amygdala, hippocampus, entorhinal/inferior temporal cortex, and neocortex; A + H + E = TDP-43 immunoreactive inclusions are present in the amygdala, hippocampus, and entorhinal/inferior temporal cortex; AD = Alzheimer disease; TDP-43 = TAR DNA-binding protein 43.

Table 2 shows the association between typicality and established neuropathologic rating scales of AD and non-AD pathologies. Most individuals showed a high ADNC at postmortem (Figure 1B). Typicality was significantly associated with Thal Aβ phase (96.8% at A3, i.e., phase 4–5; Figure 2A), neuritic plaques (87.1% at C3, i.e., frequent neuritic plaques; Figure 2C), and presence of TDP-43 inclusions (Figure 3B). These significant associations were negative, that is, a lower value of H:C (limbic-predominant AD) was associated with a higher pathologic burden or presence of pathology.

**Table 2:**
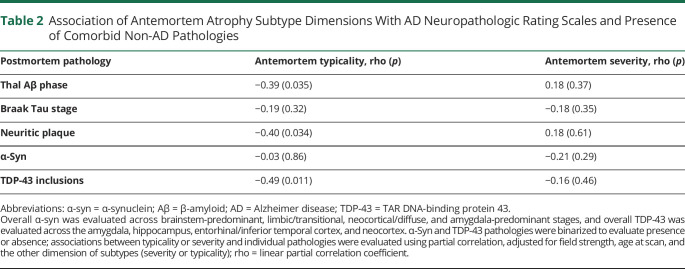
Association of Antemortem Atrophy Subtype Dimensions With AD Neuropathologic Rating Scales and Presence of Comorbid Non-AD Pathologies

**Figure 4:**
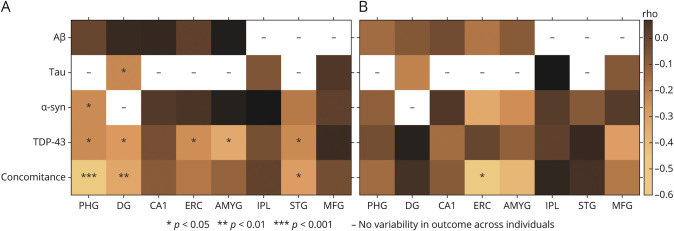
Association Between Antemortem MRI-Based (A) Typicality and (B) Severity and Regional Neuropathologic Features Associations between each of typicality or severity and presence of regional pathologies were evaluated using linear partial correlation, adjusted for field strength, age at scan, and the other dimension (severity or typicality); linear partial correlation coefficient (rho) and significant *p* values are indicated. AMYG = amygdala; Aβ = β-amyloid (diffuse and cored plaques); CA1 = hippocampus at the level of lateral geniculate nucleus including cornu Ammonis 1 subfield; DG = hippocampus at the level of lateral geniculate nucleus including dentate gyrus; ERC = entorhinal cortex; IPL = inferior parietal lobe (angular gyrus); MFG = middle frontal gyrus; PHG = hippocampus at the level of lateral geniculate nucleus including parahippocampal gyrus; STG = superior and middle temporal gyri; Tau = phosphorylated tau assessing neurofibrillary tangles; TDP-43 = phosphorylated TAR DNA-binding protein 43 neuronal cytoplasmic inclusion; α-syn = alpha-synuclein Lewy body pathology.

Figure 4A shows the association between typicality and regional neuropathologic measures. Typicality was significantly associated with the presence of (1) tau in the dentate gyrus; (2) α-syn in the parahippocampal gyrus; (3) TDP-43 in the parahippocampal gyrus, dentate gyrus, entorhinal cortex, amygdala, and superior/middle temporal gyri; and (4) concomitance of the AD and non-AD pathologies. These associations were negative, that is, a lower value of H:C (limbic-predominant AD) was associated with presence of pathology or higher concomitance of pathologies (eFigure 2–3, links.lww.com/WNL/C4).

### Association Between Antemortem Severity and Neuropathologic Outcomes

There were no significant associations between severity and established neuropathologic rating scales of AD and non-AD pathologies (Table 2).

Neither were there any significant associations between severity and regional neuropathologic measures (Figure 4B). However, severity was negatively associated with concomitance of AD and non-AD pathologies in the entorhinal cortex. This indicates that a lower value of BV:CSF showed a higher concomitance of multiple pathologies (eFigure 3, links.lww.com/WNL/C4).

### Antemortem Atrophy Subtypes and Primary and Secondary Postmortem Diagnosis

The primary neuropathologic diagnosis was ADNC in all individuals (Figure 1B). Several cases had a secondary neuropathologic diagnosis (Figure 1C), including LB disease (n = 16, 51.610%), medial temporal TDP-43 pathology and/or hippocampal sclerosis (n = 4, 12.900%), and cerebrovascular pathology (subdural hemorrhage, intracerebral hemorrhage, and/or subcortical arteriosclerotic leukoencephalopathy [n = 3, 9.690%]). Qualitatively, cases assigned to have TDP-43 in the medial temporal lobe or hippocampal sclerosis inclined toward limbic-predominant AD or typical AD. Cases assigned to have LB pathology tended to be limbic-predominant AD (dementia with LB pathology), hippocampal-sparing AD (amygdala-predominant LB pathology), or minimal atrophy AD (both forms). The single isolated cases with intracerebral hemorrhage, subdural hemorrhage, and subcortical arteriosclerotic leukoencephalopathy tended toward minimal atrophy AD.

### The Role of Sex and *APOE* Status as Mediators

Corresponding to each significant association detected, we found that neither sex nor *APOE* status was likely a mediator of the antemortem-postmortem relationship.

## Discussion

Our study investigated the relationship between antemortem atrophy subtypes and combinations of different AD and non-AD pathologies assessed postmortem. Heterogeneity in AD is a multifaceted phenomenon involving combinations of protective factors, risk factors, and concomitance of non-AD pathologies.^7^ The relative contribution of different pathologies to disease heterogeneity has been primarily reported from the postmortem (neuropathologic) perspective,^16,24-28^ with only 1 study offering an antemortem (neuroimaging) perspective,^29^ to our knowledge. Our study serves as a direct antemortem-to-postmortem investigation examining the interplay of different pathologies in atrophy subtypes of AD.

From the antemortem perspective, we treated biological heterogeneity in atrophy as continuous phenomena,^15^ that is, we examined an MRI-based operationalization of the conceptual framework for AD subtypes in terms of typicality and severity.^7^ This approach is complementary to previous studies that conventionally categorize individuals into distinct subtypes.^30-33^ We observed a nonsignificant association between typicality and severity, suggesting that disease typicality (proxied by H:C) may not be influenced by disease staging or severity (proxied by BV:CSF), thus serving as orthogonal dimensions of heterogeneity. It is important, however, to note that our initial approach of treating typicality and severity dimensions separately (while controlling for the other dimension) may be rather simplistic and deserves future exploration. This is best exemplified by cases RID 1203 and RID 1452 (Figure 1A). Despite having a lower severity (higher BV:CSF), case RID 1203 was described as hippocampal-sparing AD rather than a minimal atrophy AD. Thus, the combined contribution of typicality and severity must be factored in, that is, every individual along the typicality dimension must also be interpreted in conjunction with the corresponding severity level and vice versa.

Our key finding was that antemortem typicality, but not severity, was associated with different pathologies observed postmortem, including Aβ, tau, α-syn, and TDP-43. One reasoning for the lack of association between antemortem severity and postmortem pathologies could be that most individuals were at advanced disease stages (high ADNC), contributing to a low variability in postmortem disease severity. Below, we discuss the role of individual pathologies in relation to antemortem heterogeneity in atrophy.

We found an association between typicality and Thal Aβ stages, suggesting lower Aβ in the hippocampal-sparing AD atrophy subtype, which is consistent with a recent meta-analysis evaluating the proportion of Aβ positivity in this subtype.^7^ This result may be expected given that Aβ hallmark pathology in AD is rather diffuse, which may be indirectly associated with some degree of downstream atrophy.^34^ However, we did not find a significant association of typicality with regional ratings of Aβ density, perhaps because Aβ accumulation is usually widespread and homogeneous, with little regional specificity. To some degree, this lack of regional associations likely reflects the lack of topographical correspondence between Aβ and atrophy because evidence suggests a closer relationship between atrophy and tau than atrophy and Aβ.^35-37^

We did not observe an association between typicality or severity and Braak NFT stages, although the AD dementia cases (N = 26 at Braak stage V or VI) were at relatively more advanced stages than aMCI (N = 5 at Braak stage III or V). This lack of association is most likely because of little variability in this measure because all but 2 cases (Braak stage III, both aMCI) were at Braak stage V or VI. When assessing NFT load regionally, however, the limbic-predominant AD atrophy subtype was associated with the presence of tau pathology in the hippocampus. This is not surprising because tau pathology is a hallmark of AD affecting the hippocampus, particularly the dentate gyrus, which is known to contain the largest density of synapses.^38^ Thus, the presence of tau pathology may eventually be reflected in significant atrophy in the region, which is a key characteristic of the limbic-predominant AD atrophy subtype. Conversely, the hippocampal-sparing AD atrophy subtype was associated with the absence of tau pathology in the dentate gyrus of the hippocampus. Supporting evidence for the association between atrophy and tau pathology in this subtype is not straightforward, owing to factors including the interval between assessments of these biomarkers,^15^ regional nonspecificity of atrophy, and disagreement of subtyping methods based on these biomarkers.^39^ Altogether, our study is useful in providing a direct link between antemortem atrophy and postmortem tau pathology, suggesting that hippocampal atrophy relative to neocortical atrophy can track postmortem NFT subtypes.^16^

When assessing α-syn LB pathology regionally, we observed that the limbic-predominant AD atrophy subtype may be more prone to presence of this pathology. We also found that the parahippocampal gyrus was significantly associated with the presence of overall α-syn pathology. These findings corroborate previous postmortem neuropathologic studies showing increased α-syn pathology in typical AD and limbic-predominant AD tau subtypes.^16,24^ However, other postmortem neuropathologic studies have reported increased α-syn pathology in the hippocampal-sparing AD tau subtype.^25,26,29^ Moreover, a recent antemortem MRI study in dementia with LB observed predominance of the hippocampal-sparing atrophy subtype.^40^ It must, however, be noted that most of the postmortem studies reporting the presence of α-syn pathology to date have characterized tau subtypes, which are not necessarily interchangeable with atrophy subtypes in AD.^15,39^ Therefore, future in vivo investigations are warranted to confirm the role of α-syn pathology in AD heterogeneity. Furthermore, α-syn LB (neocortical) pathology may potentially interact with tau (Braak stage V–VI) pathology and advanced age in our cohort, explaining atrophy in the limbic-predominant AD atrophy subtype, given that limbic atrophy is not observable in the absence of these factors.^41^

Our most robust findings included the association of the limbic-predominant AD atrophy subtype with the presence of TDP-43 pathology. The limbic-predominant AD tau subtype has been described to be more prone to exhibiting TDP-43 in previous postmortem studies.^16,24,26^ It is thus plausible for the limbic-predominant AD atrophy subtype to follow suit, given the topographical similarity between tau and atrophy patterns in limbic-predominant AD.^15^ Congruent with the report from the recent meta-analysis,^7^ our study provides an antemortem-to-postmortem validation and evidence supporting the association of the limbic-predominant AD atrophy subtype with TDP-43. We observed a gradually increasing number of brain regions being affected by TDP-43 as one moves along the typicality dimension toward limbic-predominant AD. Regional examination revealed the strongest association between typicality and presence of TDP-43 in the amygdala, an initial affected site by this pathology,^28^ as well as in other medial temporal lobe structures (hippocampus and entorhinal cortex), shown to be affected by a recent antemortem study.^6^ As a main contributor of pathology affecting the hippocampus, TDP-43–associated hippocampal atrophy may be detectable at least 10 years before death.^42^ Thus, the limbic-predominant AD atrophy subtype is most likely to exhibit limbic-predominant age-related TDP-43 encephalopathy neuropathologic changes.^43^ In the absence of in vivo biomarkers assessing TDP-43, antemortem atrophy-based typicality (H:C) as a consistent correlate of postmortem TDP-43 in our study indicates the potential of this index as an antemortem proxy for this pathology.

Another main finding of our study was that both typicality and severity were regionally associated with concomitance of pathologies. This relationship was such that limbic-predominant AD and typical AD subtypes were associated with higher concomitance, whereas hippocampal-sparing AD and minimal atrophy AD subtypes were associated with lower concomitance. There also appears to be a region-specific effect, whereby some regions may accumulate a greater number of pathologies whereas other regions may be spared. For example, limbic-predominant AD was associated with a higher concomitance of different pathologies, particularly in the medial and superior temporal structures, and typical AD was associated with higher pathologic concomitance in the entorhinal cortex. Of interest, hippocampal structures including the dentate gyrus and CA1 demonstrated a generally lower concomitance than other regions. The divergent reports mentioned previously on α-syn pathology being associated with limbic-predominant and typical atrophy subtypes may be due to the higher susceptibility of the subtypes to multiple or mixed pathologies.

Finally, although qualitative, individual-level secondary postmortem diagnoses aided in providing greater confidence to our quantitative findings. Two cases with lower H:C index (toward limbic-predominant AD) were diagnosed to have TDP-43 in the medial temporal region, consistent with our main quantitative findings. Two additional cases with lower H:C index were diagnosed to have hippocampal sclerosis, which is known to correlate well with TDP-43 pathology.^16,24^ Five of 6 cases with relatively higher H:C index (toward hippocampal-sparing AD) were assigned to have amygdala-predominant LB pathology, a distinct pathologic entity.^4^ Whether/how the presence of LB pathology in the amygdala plays a role in the disposition of the hippocampal-sparing AD atrophy subtype to the pathology remains to be seen. Three cases with relatively higher H:C (toward hippocampal-sparing AD) and higher BV:CSF (toward minimal atrophy AD) indices were diagnosed with cerebrovascular pathologies. Although the lack of variability in the measure of cerebrovascular disease did not allow us to account for it in our quantitative analyses, these qualitative observations align with recent evidence, showing that cerebrovascular disease may particularly affect hippocampal-sparing AD^44^ and minimal atrophy AD subtypes.^44,45^

**Figure 5:**
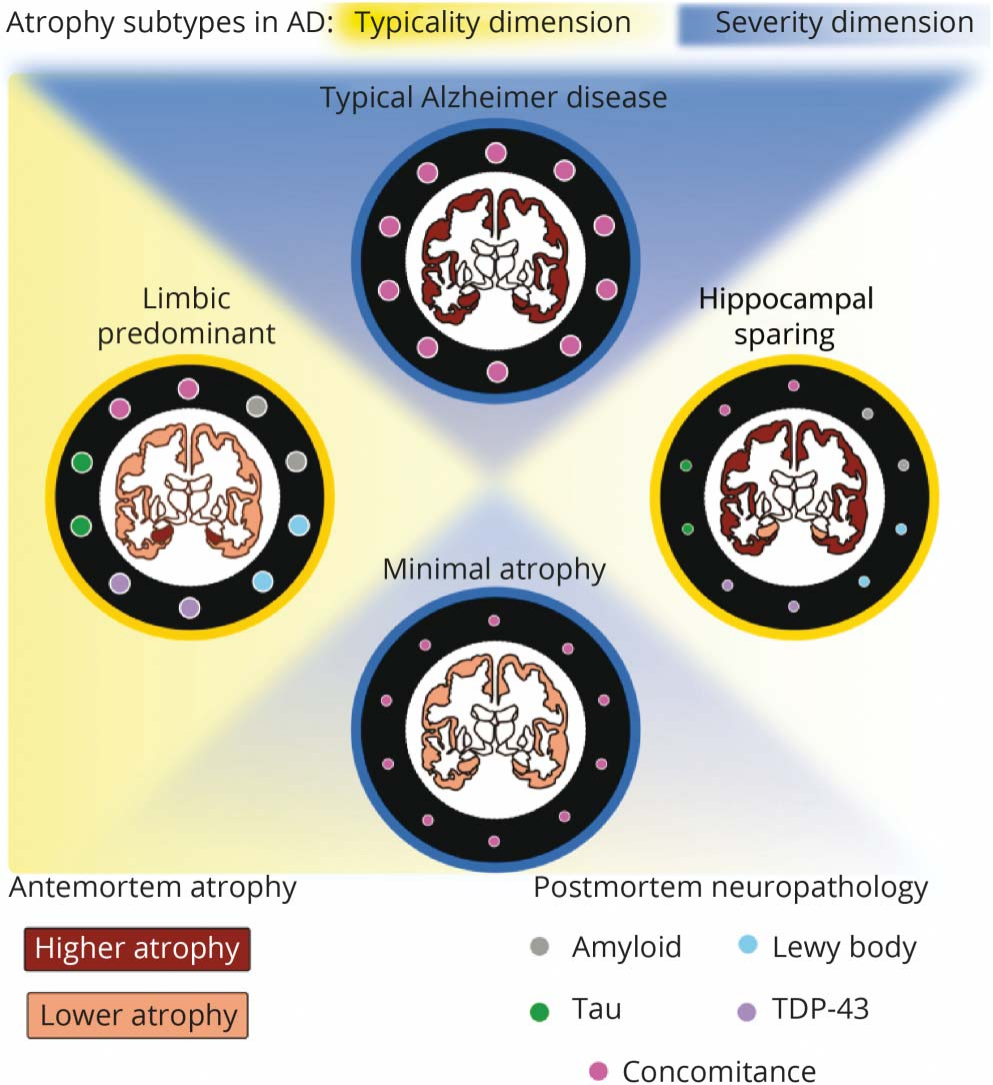
Susceptibility of Antemortem MRI-Based Heterogeneity to AD and Non-AD Neuropathologies Associations of antemortem typicality and severity with postmortem neuropathologic features may generate the following hypotheses: (1) the orthogonal dimensions of biological heterogeneity, typicality and severity, may offer complementary information regarding the vulnerability of the brain to AD (amyloid, tau) and non-AD (α-syn, TDP-43) pathologies; and (2) limbic-predominant AD along the typicality dimension and typical AD along the severity dimension may share similar underlying biological pathway(s), which make them more susceptible to pathologies, whereas hippocampal-sparing AD along the typicality dimension and minimal AD along the severity dimension may share similar pathway(s), making them less susceptible. α-syn = α-synuclein; AD = Alzheimer disease; TDP-43 = TAR DNA-binding protein 43.

Considering our current findings, we propose 2 hypotheses for future work, as larger antemortem-postmortem datasets become available (Figure 5): (1) biological heterogeneity, characterized by the orthogonal dimensions of typicality and severity, captures different aspects of vulnerability of the brain to AD and non-AD pathologies. Although typicality may be relatively more sensitive to individual pathologies varying regionally, severity may predominantly reflect a cumulative contribution of several pathologies, measured as concomitance; (2) limbic-predominant AD and typical AD subtypes may follow a unique biological pathway that tends to be affected by greater accumulation, interaction, and concomitance of various pathologies, distinct from the pathway followed by the hippocampal-sparing AD subtype that may be less affected. It is unclear which pathway the minimal atrophy AD subtype may follow: at antemortem, individuals tending toward minimal atrophy AD were at early disease stages (i.e., amnestic mild cognitive impairment) and could have eventually progressed into one of the other 3 subtypes, thus possibly following either of the 2 hypothesized pathways; at postmortem, however, many of these individuals showed high ADNC despite having minimal atrophy, suggesting that minimal atrophy AD may share the pathway common to the hippocampal-sparing AD subtype of being less affected by concomitance of various pathologies. Although our current and recent works^46,47^ provide initial support, these hypotheses need to be tested by future studies to understand their potential validity across different modalities (heterogeneity assessed by measures other than atrophy), pathologies (e.g., vascular burden), and disease stages (including predementia cases).

Our study has some limitations. First, the sample size of our cohort was limited, which may reduce the power to detect significant associations and generalize findings. However, our sample size was comparable with previous studies combining antemortem and postmortem data.^48,49^ Despite the size, we observed representation of 4 subtypes, and we chose methodologies proportionate to this limited sample size by modeling heterogeneity with continuous measures (typicality and severity) and analyzing heterogeneity using partial correlation models to maximize statistical power. Second, postmortem pathologies were only available as semiquantitative scores (i.e., gross burden of pathology), which we further binarized for the presence/absence of pathologies for sufficient statistical power. These scales may not be as sensitive as quantitative scores obtained from digital histology techniques (e.g., specific counts, density, or percentage of pathology per region). Third, most of the individuals showed a high ADNC (low variability in postmortem severity), which may have influenced the finding that the associations of antemortem MRI typicality with postmortem pathologies were stronger than those of MRI severity. Future investigations should include a broader range of pathologic severity to fully explore associations for the severity dimension. Finally, all data were sourced from the ADNI, known to have relatively strict inclusion criteria. Therefore, our current findings would need to be further validated by future studies using less restrictive and more heterogeneous cohorts.

In conclusion, we examined the relationship between antemortem MRI-based atrophy subtypes (modeled as continuous phenomena) and postmortem neuropathology in AD. In our cohort, antemortem typicality shared a stronger overall and region-specific association with different postmortem pathologies, including Aβ, tau, α-synuclein, and TDP-43, compared with antemortem severity. This suggests that the novel operationalization of biological heterogeneity in AD including typicality as a continuum is a promising proxy for the presence and regional distribution of pathologies, irrespective of disease staging (severity). Thus, factoring in contributions of core AD and comorbid non-AD pathologies toward biological heterogeneity in unspecific markers of neurodegeneration may subsequently serve as an avenue for precision medicine and future multifactorial therapies.

## Glossary

AD: Alzheimer disease
ADNC: AD neuropathologic change
ADNI: Alzheimer's Disease Neuroimaging Initiative
ADNI-EF: composite cognitive score for executive function
ADNI-MEM: composite scores for memory
aMCI: amnestic mild cognitive impairment
Aβ: β-amyloid
CDR: Clinical Dementia Rating
MMSC: Mini-Mental State Examination
NCI: neuronal cytoplasmic inclusion
NFT: neurofibrillary tangle
TDP-43: TAR DNA-binding protein 43
α-syn: α-synuclein
